# Structural Analysis of Carbon Fiber 3D-Printed Ribs for Small Wind Turbine Blades

**DOI:** 10.3390/polym14224925

**Published:** 2022-11-15

**Authors:** Víctor A. Ramírez-Elías, Noemi Damian-Escoto, Kyosung Choo, Miguel A. Gómez-Martínez, Antonio Balvantín-García, José Angel Diosdado-De la Peña

**Affiliations:** 1 Departamento de Ingeniería Mecánica, Universidad de Guanajuato, Salamanca, Guanajuato 36885, Mexico; 2 Mechanical Engineering, Youngstown State University, Youngstown, OH 44555, USA; 3 Departamento de Ingeniería Eléctrica, Universidad de Guanajuato, Salamanca, Guanajuato 36885, Mexico; 4 Material Science and Engineering, Youngstown State University, Youngstown, OH 44555, USA

**Keywords:** additive manufacturing, wind turbine ribs, delamination, short carbon fiber, PLA

## Abstract

This work provides a structural analysis of small-scale 3D-printed wind turbine ribs subjected to compression. The ribs were manufactured according to NACA 23015 and NACA 633618 geometries, with polylactic acid (PLA) and polylactic acid with carbon fiber additives (CF-PLA). In addition, holes were manufactured into the sample bodies by either 3D printing or drilling for being compared with solid samples. The compression testing was performed by following the ASTM 695D standard, whereas the beginning and propagation of delamination were assessed with the ASTM 5528 standard. Experimental results revealed that 3D-printed built-in holes provided higher compression strength, hence higher structural efficiency, than the drilled samples. Significant improvement by adding carbon fiber additives into the PLA resin system in comparison to raw PLA was detected for at least one of the studied airfoil profiles. NACA geometries also represented a key parameter for avoiding stress concentration areas, as the FEM modeling supported. However, in damaged areas, fracture mechanisms were observed such as bead-bridging, which is a key parameter in reinforcing and consolidating the specimen bodies. Working in better interphase bonding and different additives between beads and layers is highly suggested for future studies.

## 1. Introduction

The use of renewable energy systems (RES) for the generation of electricity is increasing worldwide, where natural resources such as wind energy have been classified as the most relevant [1]. Just as a reference, Mexico has a wind installed capacity surpassing 10,000 GWh with an estimation of 1700 h of useful wind per year [2,3], but this only represents around 5.0% of the contribution to the power output generation of the country [1]. It is believed that wind power has the potential to provide 20% of global electricity production by 2030, if appropriate investment is made and research and development activities are carried out [4].

The wind power industry has delved into Additive Manufacturing (AM) and multiple rapid prototyping techniques to develop small models and real-scale prototypes of wind turbine components [5,6,7,8,9,10,11,12,13]. AM, also called 3D printing, enables the fabrication of items through successive layer-upon-layer deposition, allowing the creation of intricate geometries that are overly complicated to build using traditional manufacturing processes [14,15]. The usage of AM technologies also facilitates the construction of features at several hierarchical scales, with customized material processing at different zones, and opens up a wide range of design spaces to be explored [16]; in addition, AM has been applied in diverse industries such as medical, pharmaceutical, power generation, construction, electronics, automotive, aerospace, sports, art, and fashion, among others [17,18,19,20].

AM has allowed studying structures made of diverse materials [21], through the different categories defined by the International Organization for Standardization (ISO) and the American Society for Testing and Materials (ASTM) [22]: material extrusion, material jetting, sheet lamination, vat photopolymerization, powder bed fusion, and direct energy deposition. Among these categories, fused deposition modeling (FMD), a type of material extrusion technique, is the most common method for polymeric applications [19,23], and although parts printed via FMD exhibit limited resolution, limited surface finishing, and anisotropy, this method can be carried out in low-cost equipment, in which fully functional parts can be manufactured, with multiple and customizable materials [20,24,25]. FMD, also known as fused filament fabrication (FFF), uses a continuous filament of a thermoplastic polymer that is heated to a semi-solid state and then extruded on a surface or over previously deposited layers [26,27]. Frequently, said filament is made of acrylonitrile butadiene styrene (ABS) or polylactic acid (PLA), but other thermoplastics are commonly used, such as acrylonitrile styrene acrylate (ASA), polycarbonate (PC), polyetherimide, high-impact polystyrene (HIPS), thermoplastic polyurethane (TPU), aliphatic polyamides (PA, nylon), polyethylene terephthalate (PET), polyvinyl alcohol (PVA), and high-performance plastics such as polyether ether ketone (PEEK) and polyetherimide (PEI), among others [18,25,28,29,30].

Aggregates in polymeric filaments were introduced to improve the performance of 3D-printed parts in terms of mechanical strength, thermal conductivity, electrical conductivity, fire-retardant tendency, piezoelectric properties, or magnetic properties [31]. These additively manufactured composites (AMCs) are classified by matrix and reinforcement type; particularly, polymer matrix composites (PMCs) can be reinforced with particles, fibers, and nanostructures [32,33,34]. The properties of any AMC can be customized by means of the aggregate volume fraction to meet specific requirements of the material, which has been addressed by several researchers [35]. On the other hand, research is required to reduce porosity and other defects, as well as postprocessing 3D-printed components [36].

Several defects have been studied in laminate composites, where the most common defects are resin cracking, fiber breaking, and delamination [37,38]. The latter has also been identified as a common defect for 3D printed parts [39,40], which negatively influences the performance of the component. Delamination in test samples manufactured by FDM has been studied for diverse loading conditions, such as by Garg and Bhattacharya [41] who printed and tested ABS tensile specimens, based on the ASTM D638 standard, concluding that stress is concentrated at the narrow zone in and around the intra-layer bond region which causes the failure of the specimen due to delamination of layers from the bonded region. Mercado-Colmenero et al. [42] analyzed polyethylene terephthalate glycol (PETG) under uniaxial compression loads according to the ISO-604 standards, where the test specimens manufactured along the z-axis presented brittle fracture caused by a delamination process. Barile et al. [43] studied the effect of the extrusion temperature on the interlayer cohesion properties of ABS parts by employing a double cantilever beam (DCB) test according to ASTM D5528 in combination with Acoustic Emission (AE) to determine the delamination energy GI (Mode I). Results showed that increasing the extrusion temperature causes reduced delamination with respect to a large deformation phase, inducing an increase in the GI toughness. This was also concluded by Khan et al. [44], who included Mode II and mixed-mode loadings reaching similar conclusions.

Similar tests have been used for analyzing AMCs. For instance, Heidari-Rarani et al. [45] performed a comparative study of tensile and bending specimens printed with pure PLA and continuous carbon fiber reinforced PLA (CCFR-PLA). The tensile test for pure PLA and CCFR-PLA followed ASTM D638 and ASTM D3039 standards, respectively, while the bending test followed the ASTM D790 standard. Bending results showed that the first micro-cracks occurred between the layers and propagated along the specimen length, which caused delamination between the two layers. The authors assumed that when the interlaminar cracks reach each other, they create vertical cracks in the layer just below the loading nose; in addition, they concluded that the dominant failure modes of CCFR-PLA are delamination and delamination-induced matrix cracking. Ghebrentinsae et al. [46] built tensile and flexural tests samples following ASTM D3039 and ASTM D7264-07 standards where a carbon fiber (CF) filament and Markforged^®^ Onyx were used. The experimental work showed delamination failure in both flexural and tensile test samples; it was observed between the matrix and fiber layers and the matrix material was easy to peel off from the fibers. The authors attributed the delamination to weak bonding between successive layers. Somireddy et al. [47] investigated the mechanical behavior of 3D-printed ABS with short carbon fiber (SCF) reinforcements by conducting tensile tests based on ASTM D3039 and ASTM D5528 for interlaminar fracture toughness of the parts under the crack opening mode. The authors of [47] concluded that the presence of voids in the extrudates and into the layer interfaces causes delamination, which along with the fracture of SCFs, degraded the overall material properties of the printed parts. Maqsood and Rimašauskas [48] combined short carbon PLA with continuous carbon fiber (CCF) to form CCFR thermoplastic composite (CCFRTC) material and built specimens for an ASTM D790 standard flexural test. During bending, delamination occurred towards the sides of the applied load between the layers instead of breakage or fracture in the composite part, starting from the upper layer lines and shifting towards the bottom lines.

Controlling delamination in 3D-printed parts via FDM has been explored by means of special material formulations, such as Chen et al. [49] who were able to eliminate layer delamination by formulating a bisphenol A-based epoxy and benzoxazine with carbon nanotubes (CNT) to a low-temperature thermoplastic, which requires a high-temperature curing process. This resulted in a covalently cross-linked material, where the cross-layer reaction fuses individual layers, producing flexural and tensile test samples with similar behavior, regardless of the infill orientation. Other researchers have opted for built-in features, such as Sanei et al. [50] who studied the effects of printed discontinuities on ASTM D-3039 tensile test samples using SFC nylon and continuous carbon fiber (CCF) reinforcements around the periphery of an open hole. Test results showed damage on the periphery of the hole as fiber–matrix debonding, but failure propagated around and not into the hole. The authors of [50] concluded that with 3D printing there is no delamination or damage induced in the creation of the hole, and that it can be used to strengthen the parts at the discontinuity region to mitigate the effect of stress concentrations.

Although specific defects have been studied in 3D-printed standardized test samples manufactured through FDM, and the literature also reports how some special features improved the performance of said samples, information is limited on this topic regarding additively manufactured functional models, their performance under different loading conditions, as well as the effects of geometric modifications on them, either built-in or post-processed through machining [51]. In addition, in spite of the evidence that AM is a suitable means to build small wind turbine blades and components with diverse materials [5,6,10,11,12], including standardized blade profiles such as NACA 0015 [13] and SG6043 [9], there is a reduced amount of data of the structural performance of such 3D-printed elements. Therefore, the aim and novelty of this work is to present an experimental structural analysis of components for small wind turbines, particularly the ribs that shape the turbine blades, built through FDM with two commercially available polymers, including SCF additive reinforcements. Ribs were designed and manufactured with airfoil geometries, specifically NACA 23015 and NACA 633618, and their performance under compressive load was assessed. Initially, a simple solid specimen, to characterize the elastic behavior and ultimate strength of the material, was used and defined as a control case. These kinds of components are used in lightweight structures, and for this reason often they might have a specific perforation pattern used as free spaces where wires, pipes, or hydraulic systems can pass throughout; in addition, near the center of gravity, they count with circular or square holes at which central beams are assembled, and simultaneously the holes are used as weight-saving features. Therefore, the effects of geometrical features, built-in holes and drilled holes, are analyzed herein and compared with the control case. The testing procedure involved compression loading of the 3D-printed components until failure, and micrographs of the damage were performed in order to determine if delamination may be greater and could propagate faster by holes originally 3D printed or by conventionally drilled holes. Finally, finite element analysis (FEA) of the ribs is included to complement the proposed comparative study. The following section details the materials and equipment used to build and test the specimen sets, along with the experimental procedures and a description of the analyses carried out. Results and discussion of the experiments and analyses are presented in Section 4, followed by the Conclusions section.

## 2. Materials and Methods

In this work, the raw materials used were PLA (polylactic acid) filaments (Ø = 3 mm) supplied by Makermex^®^ (León, Mexico), and PLA filaments (Ø = 3 mm) with carbon fiber additives (20% *w*/*w*), Proto-Pasta CFD12805, provided by Shenzhen Hanwei Technology Co. Ltd. (Shenzhen, China). According to the literature, the mechanical properties for these similar filaments are listed in Table 1.

**Figure 1:**
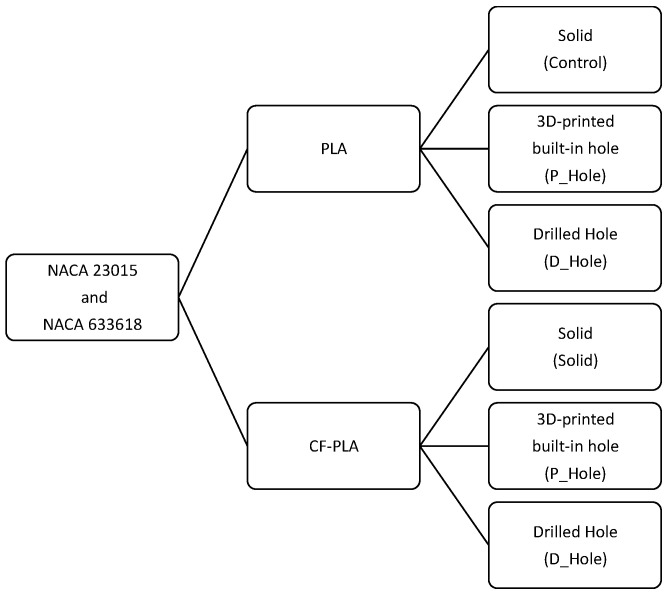
Sample experimentation plan.

With the purpose of assessing the compression strength and consequent delamination of 3D-printed rib specimens, a set of different samples was designed for comparing different features that may be involved in the propagation of delamination when a certain 3D-printed rib specimen is subjected to compression. The first approach is to assess the compression strength of a simple solid specimen to characterize the elastic behavior and ultimate strength that will be used as a control case. As mentioned above, ribs with airfoil profiles usually have various features used as free spaces for wiring or hydraulic systems, in addition to the holes near the gravity center. The airfoil profiles considered for the set of rib samples were NACA 23015 and NACA 633618, which are airfoil geometries often used in aerodynamical structures for wind turbine blades. These airfoils were 3D printed with PLA and PLA with short carbon fiber additives (CF-PLA), and eventually each of the sets was sorted as solid (control), originally 3D-printed built-in hole (P_Hole), and machined with a drilled hole (D_Hole), as Figure 1 shows.

**Figure 2:**
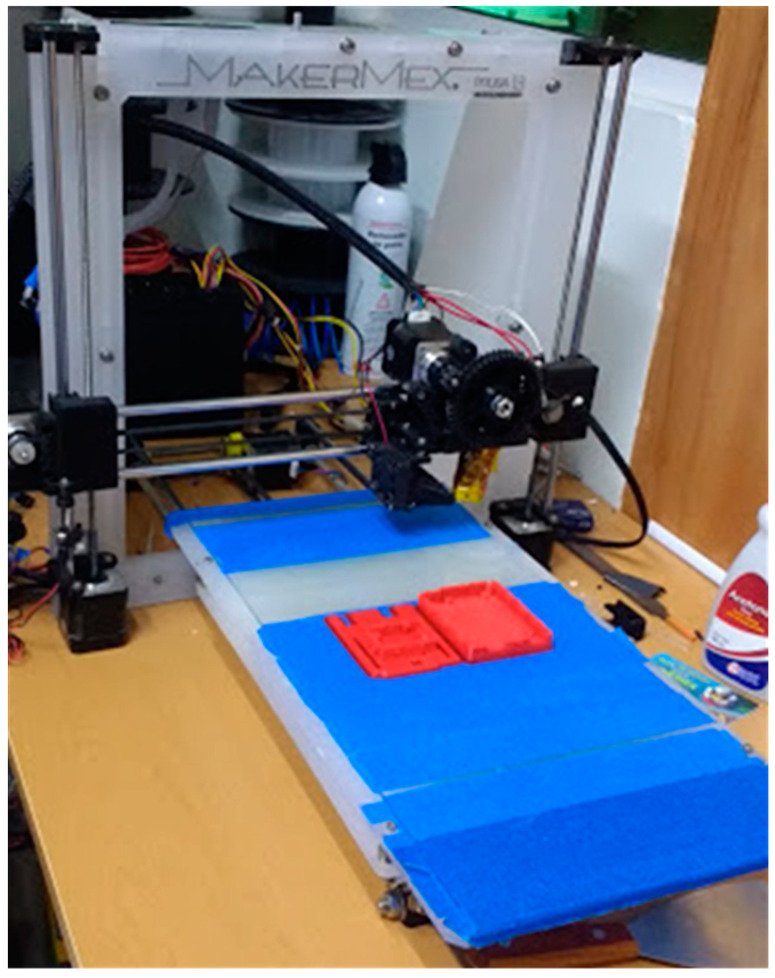
Prusa^®^ 3D printer.

All the samples shown in Figure 1 are composed of 10 specimens per sample and they were manufactured in a Prusa^®^ 3D printer (Prague, Czech Republic) (see Figure 2). The samples were all designed with 100 mm length, 15 mm width, and a thickness of 5 mm; in addition, specifically for the P_Hole samples, a central hole (Ø = 10 mm) was also included. Once the CAD models of the samples were defined, they were processed with Cura^®^ Slicer (Utrecht, The Netherlands), in which other parameters were also determined as Table 2 lists.

**Figure 3:**
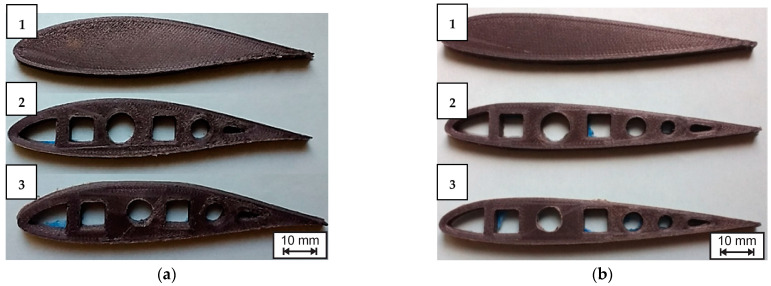
Examples of different simples manufactured for this work: (**a**) NACA 633618, CF-PLA; (**b**) NACA 23015, CF-PLA; 1—solid (control); 2—P_Hole; 3—D_Hole.

For the D_samples (without any printed hole), central holes were machined by drilling with a 10 mm drill bit and feed speeds of 100 rpm for PLA samples and 200 rpm for CF-PLA samples. Cooling water was used to stop any significant increase in temperature during the drilling process. The samples were classified and finished by deburring rough edges, as Figure 3 shows. Finally, each sample was weighed with a Vibra^®^ Laboratory Balance (DeSoto, TX, USA).

### 2.1. Testing and Data Processing

**Figure 4:**
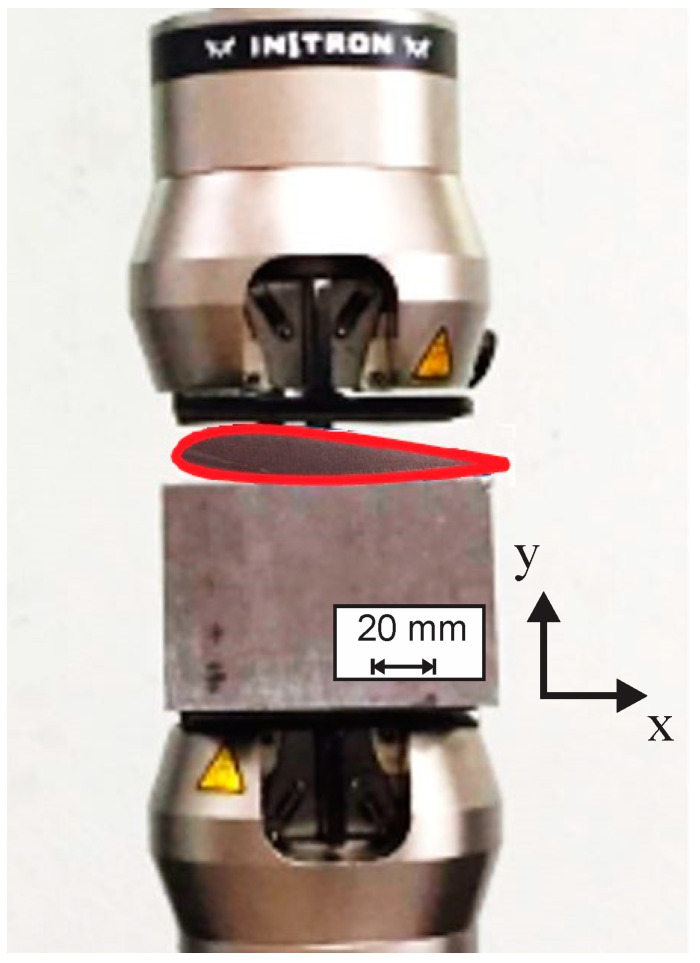
Specimen subjected to compression by the universal testing machine INSTRON 8872.

With the purpose of simulating typical behaviors and damages that the ribs show in a certain lightweight structure, it was determined that compression loads along the ordinate axis are the most predominant [54,55]. Thus, a typical compression test for rigid plastics was carried out by following the ASTM 695D standard and using a universal testing machine INSTRON 8872, with a loading cell of 100 kN. A simple steel fixture was manufactured to fix the specimens in a horizontal position to be loaded along the ordinate axis, as Figure 4 shows; a compression load was applied at 0.1 mm min-1 up to a maximum peak before a decay that suggests catastrophic internal damage.

The raw data were recorded by the machine control software Bluehill 3^®^ (Norwood, MA, USA) and graphs of load against extension per specimen were plotted. When the experimentation stage was completed, the reduction in raw data had taken place. 

**Figure 5:**
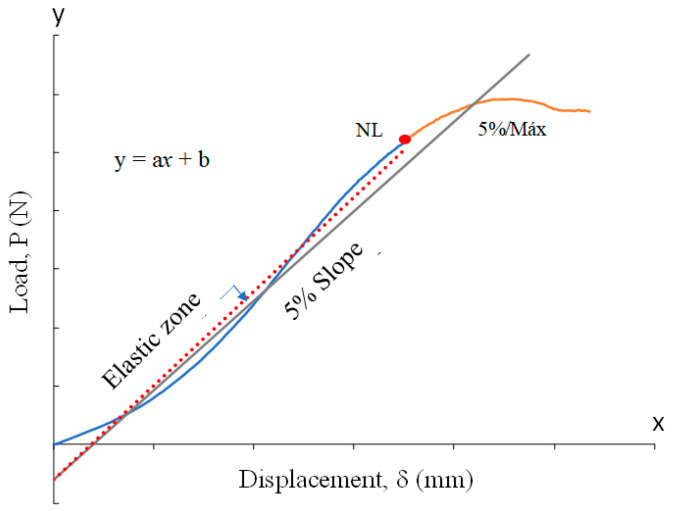
Measurements in raw data plots.

One of the innovations that this work offers is that the propagation of fractures was partially calculated by following the ASTM 5528 standard [56], which measures the Mode I interlaminar fracture toughness for laminated composite materials, as Figure 5 shows. This consisted of drawing an approximately right line throughout the elastic zone from the non-linear point (NL) or the point where the linearity is no longer evident to the origin or the interception with the ordinate axis; this line is represented by regression with a general equation, as Equation (1) shows:

(1) $$y_{1}=ax+b$$

where *y*_1_ is the lineal regression curve, a is the slope or rate between *P/δ* (N mm^−1^), and b is the interception with the ordinate axis when x = 0 (N). Subsequently, an offset line is drawn by taking 95% of the slope from the first elastic line and is projected beyond the interception of the raw data curve, as Equation (2) describes:

(2) $$y_{2}=a*0.95*x+b$$

The interception of the second line and the raw data curve is named the 5%/max point. The threshold between these two points represents the raising of an initial crack up to its propagation to become delamination [57]. When a maximum peak is found within this threshold then it is considered that the propagation runs catastrophically, as the ASTM 5528 standard suggests. In this fashion, an interlaminar initial crack and its subsequent propagation is estimated through experimental data even when visual detection is not yet possible.

### 2.2. Structural Efficiency

Structural efficiency is defined here as a ratio of the load-carrying ability of a structure to its mass. This parameter is a simplification of a structure strength as a result of its materials, geometry, joint assembly, and loading direction, either static or dynamic loading. For instance, Jegley et al. [58] applied this parameter to carbon-epoxy tapered struts to space- and aircraft under compression and tension loads, which is defined as follows [59]:

(3) $$Structural\;effieciency=\frac{Compression\;Load}{Weight\;of\;structure},$$

### 2.3. Finite Element Analysis

The distribution of stress along the specimens was modeled by FEA via ANSYS^®^ APDL (Canonsburg, PA, USA). The NACA geometries were drawn by CAD and exported into ANSYS^®^, to be meshed with SOLID187 elements. The samples were modeled with an elastic and isotropic material with PLA and CF_PLA properties (see Table 1). Finally, the compressive loading was applied at the upper surface and the lower surface was constrained in all the degrees of freedom (DoF). Once the FEA models were solved, the von Misses stresses were deployed for structural analysis.

### 2.4. Fractography

A Sinowon^®^ (Dongguan, China) metallographic microscope with 100×/400× magnification was used to obtain images of the hole cross-section after testing in order to detail the inner delamination in damaged samples. 

## 3. Results and Discussion

**Figure 6:**
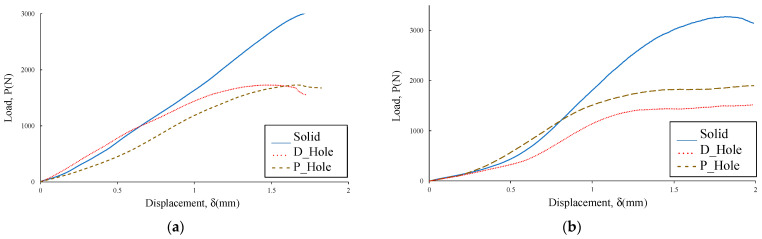
Typical experimental curves for NACA 23015: (**a**) PLA; (**b**) CF-PLA.

Once the raw data were recorded, curves per specimen were plotted and the NL point and the 5%/max point were determined. For instance, Figure 6 shows typical plots for each airfoil NACA 23015 sample. As it is observed, the slopes of each sample are consistent, however, the maximum linear peak (NL) is different, and in some cases, the non-linear peak is slightly diffuse, as the plot describes a long curve. In general terms, the solid sample shows a significantly higher NL point in comparison to the D_Hole and P_Hole, as expected, given that this sample has no deleterious holes that decrease the compression strength, though this sample shows similar elastic behavior properties to the others. As observed in the case of the PLA sample, the D_Hole sample appears to be higher than the P_Hole sample in contrast with the CF_PLA, where the P_Hole sample is clearly higher than the D_Hole sample. This change in behavior between samples might be attributable to the effect of drilling, hence abrasion and heat on the resin additives that apparently are more susceptible to interlayer damages.

**Figure 7:**
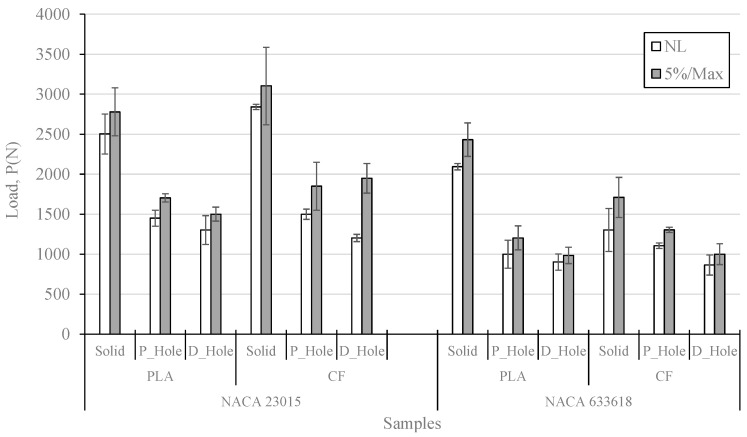
Comparison of maximum compression loading values, C.I. 95%.

Figure 7 shows the comparison of NL and 5%/max values for all the studied samples, where each bar represents a certain average value, and its respective error bar represents a 95% confidence interval. As expected, the solid samples for both types of airfoils show higher maximum loading values with a clear preponderancy by the NACA 23015, and this trend is also valid for all the remaining samples. Thus, the type of geometry might have an effect on the distribution of stresses along the specimen, with areas with higher accumulation of stresses by the NACA 633618. In general terms, the carbon fiber additives induced an increase in NL and 5%/max values in all the NACA 23015 samples, whereas this trend is not too clear for the NACA 633618. However, it is obvious that a drilling manufacturing process induces higher delamination damages than a hole manufactured by 3D printing, according to the data shown by all the samples, as mentioned above. The differences between NL and 5%/max are higher for NACA 23015 (≈350 N) than for NACA 633618 (≈220 N), which suggests that in the first airfoil the propagation of delaminations needed more compression energy to be carried out; this is consistent with a greater distribution of stresses along the specimens, as previously described. This propagation of delamination appears to be more unstable for CF_PLA samples, as they show higher variabilities; thus, it can be implied that while the carbon fiber additives induce higher reinforcement for fracture propagation, these additives also induce instabilities in such propagations, and this is noticeable in the solid samples of both airfoils.

**Figure 8:**
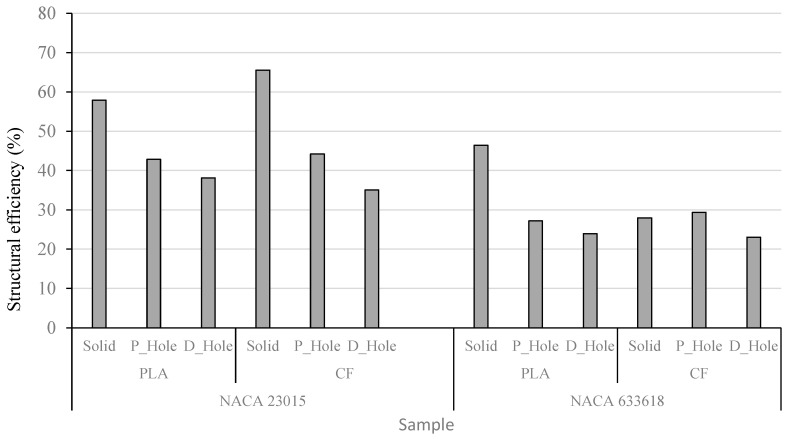
Comparison of structural efficiency values.

Among those samples with a hole, the NACA 23015-CF_PLA-P_Hole sample showed the best reinforcement either on an initial crack or propagation of delaminations, in contrast with the NACA 633618-PLA-D_Hole sample, which showed lower values. When the efficiency is determined as is shown in Figure 8, it is clear that the P_samples had the higher efficiency after the solid samples, and then it can be implied that the 3D-printed holes definitely provided better reinforcement than the drilled holes. However, the effect of the carbon fiber additives is not quite clear across all sets studied.

**Figure 9:**
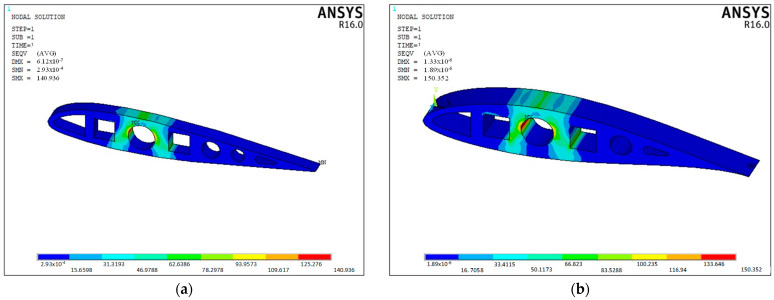
Von Mises stress distribution on PLA samples: (**a**) NACA 23015; (**b**) NACA 633618.

Once the experimental data provided maximum loading values per sample, as shown in Figure 7, these loading values were used to obtain the distribution of stress from the FEA models. As observed in Figure 9, the main distribution of stresses is located around the circular central hole, with special concentration into the inner surface with values around 140 and 150 MPa, respectively, wherein it is expected the main damages and delaminations might appear. Most of the stresses are absorbed by the material between the central circular hole and the squared holes, which suggests that this is a key parameter to reinforce the compression strength by adding more material to the original design.

**Figure 10:**
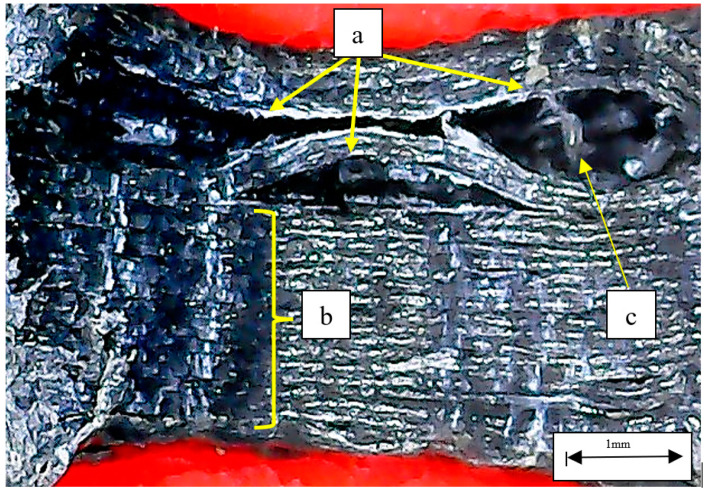
Central hole cross-section of NACA 633618 -CF_PLA-D sample: (**a**) delamination at the central hole; (**b**) damages caused by drilling; (**c**) bead-bridging mechanism.

**Figure 11:**
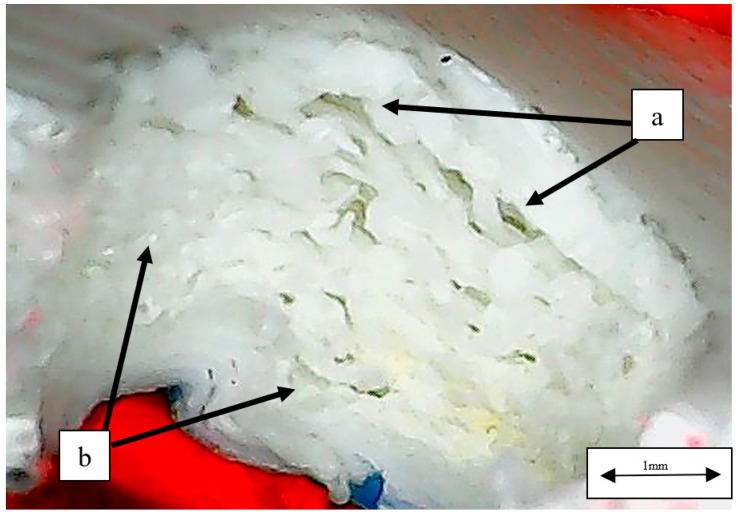
Central hole cross-section of NACA 23015-PLA-D sample: (**a**) delamination at the central hole; (**b**) damages caused by drilling and possible voids.

Fractographic images exposed some typical compression fracture mechanisms in the damaged samples. Figure 10 shows the central hole cross-section of an NACA 633618-CF_PLA-D sample where marks of drilling are observed, and such damages induce micro delamination in the material that tend to propagate, causing longer delamination when samples are compressed. Though most of the material looks consolidated, these longer delaminations are easily observed, in which a type of bead-bridging mechanism that somehow provides interlayer reinforcement is also evident. In an NACA 23015-PLA-D sample, voids and defects from the 3D manufacturing in addition to damages due to drilling are rather evident, as seen in Figure 11. These defects and damages, given their high amount, significantly degrade the material consolidation so much that it looks like a spongy material, causing high variability in compression strength values and elastic behavior, as exposed by the data above. The bead-bridging mechanism is barely observable (Figure 11a) and apparently is substituted by voids, and this might be attributable also to poor bonding between layers which, as it is obvious, is deleterious for structural applications.

## 4. Conclusions

This work presented a description of the relevance of AM in the wind power industry, as well as a summary of standard tests for the mechanical characterization of 3D-printed standardized tests samples. Along with the described tests, similarities in the behavior and analysis of composite laminates and 3D-printed parts were discussed, which led to identifying an area of opportunity for research on the characterization and defect analysis of 3D-printed functional models. Therefore, an experimental assessment of rib samples with NACA 23015 and 633618 airfoil geometries subjected to compression was presented herein. The samples were 3D printed with PLA and CF-PLA, and an experimental plan was designed for samples with a central hole made by 3D printing or machining. Subsequently, these samples were compared with samples without any hole, determined as the control case.

Once the data were analyzed, it was observed that samples with a hole showed lower strength compression values than the control sample, as expected. Among the holed samples, those with holes made by drilling showed lower mean compression strength values and higher variability than the 3D-printed built-in hole samples, which is attributable to damages and micro delamination induced by the machining process. Regarding the presence of carbon fiber additives, these did not represent a significant improvement in terms of compression strength values for all the sample sets (corroborated by Student′s *t*-test, *t* < 2.093 ∴ Ho: ${\bar{x}}_{1}={\bar{x}}_{2}$ cannot be rejected); however, the bead-bridging mechanism, observed in most of the delaminated samples, was even more evident in FC_PLA samples, as supported by fractography. This might be the cause of why these samples showed shorter delamination and better layer consolidation than the PLA samples. 

In addition, it was observed that the NACA 23015 model displayed fewer stress concentration areas, with a better strain energy distribution around the airfoil body, as FEA modeling images support.

Based on this work, it is suggested to explore other types of additives in PLA airfoil profiles that might keep improving the layer consolidation with better distribution of strain energy along the body structure. 

## Figures and Tables

**Table 1:** Mechanical properties of PLA filaments [52,53].

Mechanical Properties	PLA	CF-PLA	Units
Young’s modulus	3420	4791	MPa
Poisson	0.33	0.40	--
Tensile strength	35.6	47.9	MPa
Elongation at break	4.2	2.0	%
Flexural strength	85.2	114	MPa
Flexural modulus	2378	6320	MPa
Density	1.24	1.29	g cm^−3^

**Table 2:** The 3D printing parameters.

Parameter	PLA	CF-PLA	Units
Printing density	50	50	%
Printing temperatures	195	210	°C
Initial printing temperature	185	200	°C
Final printing temperature	180	195	°C
Filament diameter	2.85	2.85	mm
Printing velocity	50	50	mm s^−1^

## Data Availability

Not applicable.

## References

[1] Hernández-Escobedo Q., Perea-Moreno A.J., Manzano-Agugliaro F. (2018). Wind Energy Research in Mexico. Renew Energy.

[2] Gallardo R.P., Ríos A.M., Ramírez J.S. (2020). Analysis of the Solar and Wind Energetic Complementarity in Mexico. J. Clean. Prod..

[3] Hernández-Escobedo Q., Manzano-Agugliaro F., Zapata-Sierra A. (2010). The Wind Power of Mexico. Renew. Sustain. Energy Rev..

[4] Darwish A.S., Al-Dabbagh R. (2020). Wind Energy State of the Art: Present and Future Technology Advancements. Renew. Energy Environ. Sustain..

[5] Chiriță A.P., Bere P.P., Rădoi R.I., Dumitrescu L. (2019). Aspects Regarding the Use of 3D Printing Technology and Composite Materials for Testing and Manufacturing Vertical Axis Wind Turbines. Mater. Plast..

[6] Sivamani S., Nadarajan M., Kameshwaran R., Bhatt C.D., Premkumar M.T., Hariram V. (2020). Analysis of Cross Axis Wind Turbine Blades Designed and Manufactured by FDM Based Additive Manufacturing. Materials Today: Proceedings.

[7] Murdy P., Dolson J., Miller D., Hughes S., Beach R. (2021). Leveraging the Advantages of Additive Manufacturing to Produce Advanced Hybrid Composite Structures for Marine Energy Systems. Appl. Sci..

[8] Singh U., Lohumi M.K., Kumar H. (2020). Additive Manufacturing in Wind Energy Systems: A Review. Smart Innov. Syst. Technol..

[9] Porto H.A., Fortulan C.A., Porto A.J.V., Tsunaki R.H. (2022). Geometric Analysis of Small Wind Turbine Blades Manufactured by Additive Manufacturing. J. Aerosp. Technol. Manag..

[10] Rouway M., Tarfaoui M., Chakhchaoui N., Omari L.E.H., Fraija F., Cherkaoui O. (2021). Additive Manufacturing and Composite Materials for Marine Energy: Case of Tidal Turbine. 3d Print. Addit. Manuf..

[11] Davis M.S., Madani M.R. 3D-Printing of a Functional Small-Scale Wind Turbine. Proceedings of the 2018 6th International Renewable and Sustainable Energy Conference (IRSEC).

[12] Poole S., Phillips R. Rapid Prototyping of Small Wind Turbine Blades Using Additive Manufacturing. Proceedings of the 2015 Pattern Recognition Association of South Africa and Robotics and Mechatronics International Conference (PRASA-RobMech).

[13] Bassett K., Carriveau R., Ting D.S.K. (2015). 3D Printed Wind Turbines Part 1: Design Considerations and Rapid Manufacture Potential. Sustain. Energy Technol. Assess..

[14] Vafadar A., Guzzomi F., Rassau A., Hayward K. (2021). Advances in Metal Additive Manufacturing: A Review of Common Processes, Industrial Applications, and Current Challenges. Appl. Sci..

[15] Babu S.S., Goodridge R. (2015). Additive Manufacturing. Mater. Sci. Technol..

[16] Rosen D. (2014). Design for Additive Manufacturing: Past, Present, and Future Directions. J. Mech. Des..

[17] Ligon S.C., Liska R., Stampfl J., Gurr M., Mülhaupt R. (2017). Polymers for 3D Printing and Customized Additive Manufacturing. Chem. Rev..

[18] Saleh Alghamdi S., John S., Roy Choudhury N., Dutta N.K. (2021). Additive Manufacturing of Polymer Materials: Progress, Promise and Challenges. Polymers.

[19] Ngo T.D., Kashani A., Imbalzano G., Nguyen K.T.Q., Hui D. (2018). Additive Manufacturing (3D Printing): A Review of Materials, Methods, Applications and Challenges. Compos. Part B Eng..

[20] Gao W., Zhang Y., Ramanujan D., Ramani K., Chen Y., Williams C.B., Wang C.C.L., Shin Y.C., Zhang S., Zavattieri P.D. (2015). The Status, Challenges, and Future of Additive Manufacturing in Engineering. Comput. Aided Des..

[21] Thompson M.K., Moroni G., Vaneker T., Fadel G., Campbell R.I., Gibson I., Bernard A., Schulz J., Graf P., Ahuja B. (2016). Design for Additive Manufacturing: Trends, Opportunities, Considerations, and Constraints. CIRP Ann. Manuf. Technol..

[22] Additive Manufacturing Technical Committee (2015). ISO/ASTM 52900: Additive Manufacturing—General Principles—Terminology.

[23] Dudek P. (2013). FDM 3D Printing Technology in Manufacturing Composite Elements. Arch. Metall. Mater..

[24] Lee J.Y., An J., Chua C.K. (2017). Fundamentals and Applications of 3D Printing for Novel Materials. Appl. Mater. Today.

[25] Daminabo S.C., Goel S., Grammatikos S.A., Nezhad H.Y., Thakur V.K. (2020). Fused Deposition Modeling-Based Additive Manufacturing (3D Printing): Techniques for Polymer Material Systems. Mater. Today Chem..

[26] Beaman J.J., Bourell D.L., Seepersad C.C., Kovar D. (2020). Additive Manufacturing Review: Early Past to Current Practice. J. Manuf. Sci. Eng. Trans. ASME.

[27] Zhai Y., Lados D.A., Lagoy J.L. (2014). Additive Manufacturing: Making Imagination the Major Limitation. JOM.

[28] Patel R., Desai C., Kushwah S., Mangrola M.H. (2022). A Review Article on FDM Process Parameters in 3D Printing for Composite Materials. Mater. Today Proc..

[29] Ramesh M., Niranjana K., AlMangour B., Abdul bin Majid M.S. (2022). Effect of Process Parameters on Fused Filament Fabrication Printed Composite Materials. High-Performance Composite Structures. Composites Science and Technology.

[30] Cantwell W.J., Morton J. (1992). The Significance of Damage and Defects and Their Detection in Composite Materials: A Review. J. Strain Anal. Eng. Des..

[31] Bhuvanesh Kumar M., Sathiya P. (2021). Methods and Materials for Additive Manufacturing: A Critical Review on Advancements and Challenges. Thin-Walled Struct..

[32] Velu R., Raspall F., Singamneni S. (2018). 3D Printing Technologies and Composite Materials for Structural Applications.

[33] Blanco I. (2020). The Use of Composite Materials in 3d Printing. J. Compos. Sci..

[34] Bhawal P., Das K., Ganguly S., Mondal S., Ravindren R., Nc D. (2017). Fabrication of Light Weight Mechanically Robust Short Carbon Fiber/Ethylene Methyl Acrylate Polymeric Nanocomposite for Effective Electromagnetic Interference Shielding. J. Polym. Sci. Appl..

[35] Das T.K., Ghosh P., Das N.C. (2019). Preparation, Development, Outcomes, and Application Versatility of Carbon Fiber-Based Polymer Composites: A Review. Adv. Compos. Hybrid Mater..

[36] Nugroho W.T., Dong Y., Pramanik A. (2021). 3D Printing Composite Materials: A Comprehensive Review.

[37] Abbas S., Li F., Qiu J. (2018). A Review on SHM Techniques and Current Challenges for Characteristic Investigation of Damage in Composite Material Components of Aviation Industry. Mater. Perform. Charact..

[38] Nevadunsky J.J., Lucas J.J., Salkind M.J. (1975). Early Fatigue Damage Detection in Composite Materials. J. Compos. Mater..

[39] Solomon I.J., Sevvel P., Gunasekaran J. (2020). A Review on the Various Processing Parameters in FDM. Materials Today: Proceedings.

[40] Blaj M.I., Zaharia S.M., Pop M.A., Oancea G. (2022). Tensile Properties and Manufacturing Defectives of Short Carbon Fiber Specimens Made with the FDM Process. Mater. Plast..

[41] Garg A., Bhattacharya A. (2017). An Insight to the Failure of FDM Parts under Tensile Loading: Finite Element Analysis and Experimental Study. Int. J. Mech. Sci..

[42] Mercado-Colmenero J.M., Dolores La Rubia M., Mata-Garcia E., Rodriguez-Santiago M., Martin-Doñate C. (2020). Experimental and Numerical Analysis for the Mechanical Characterization of Petg Polymers Manufactured with Fdm Technology under Pure Uniaxial Compression Stress States for Architectural Applications. Polymers.

[43] Barile C., Casavola C., Cazzato A. (2019). Acoustic Emission Analysis on Mode I Delamination Tests of Fused Deposition Modelling Parts. Conference Proceedings of the Society for Experimental Mechanics Series.

[44] Khan A.S., Ali A., Hussain G., Ilyas M. (2021). An Experimental Study on Interfacial Fracture Toughness of 3-D Printed ABS/CF-PLA Composite under Mode I, II, and Mixed-Mode Loading. J. Thermoplast. Compos. Mater..

[45] Heidari-Rarani M., Rafiee-Afarani M., Zahedi A.M. (2019). Mechanical Characterization of FDM 3D Printing of Continuous Carbon Fiber Reinforced PLA Composites. Compos. B Eng..

[46] Ghebretinsae F., Mikkelsen O., Akessa A.D. (2019). Strength Analysis of 3D Printed Carbon Fibre Reinforced Thermoplastic Using Experimental and Numerical Methods. IOP Conference Series: Materials Science and Engineering.

[47] Somireddy M., Singh C.V., Czekanski A. (2020). Mechanical Behaviour of 3D Printed Composite Parts with Short Carbon Fiber Reinforcements. Eng. Fail. Anal..

[48] Maqsood N., Rimašauskas M. (2021). Delamination Observation Occurred during the Flexural Bending in Additively Manufactured PLA-Short Carbon Fiber Filament Reinforced with Continuous Carbon Fiber Composite. Results Eng..

[49] Chen Q., Han L., Ren J., Rong L., Cao P., Advincula R.C. (2020). 4D Printing via an Unconventional Fused Deposition Modeling Route to High-Performance Thermosets. ACS Appl. Mater. Interfaces.

[50] Sanei S.H.R., Arndt A., Doles R. (2020). Open Hole Tensile Testing of 3D Printed Continuous Carbon Fiber Reinforced Composites. J. Compos. Mater..

[51] Batista M., Piñero D., Ramírez M., Mayuet P.F., Bienvenido R., Vazquez J.M. (2021). Defectology Characterization of FDM Drilled Parts. IOP Conference Series: Materials Science and Engineering.

[52] Reverte J.M., Caminero M. (2020). ángel; Chacón, J.M.; García-Plaza, E.; Núñez, P.J.; Becar, J.P. Mechanical and Geometric Performance of PLA-Based Polymer Composites Processed by the Fused Filament Fabrication Additive Manufacturing Technique. Materials.

[53] Ferreira R.T.L., Amatte I.C., Dutra T.A., Bürger D. (2017). Experimental Characterization and Micrography of 3D Printed PLA and PLA Reinforced with Short Carbon Fibers. Compos. B Eng..

[54] Bottasso C.L., Campagnolo F., Croce A., Tibaldi C. (2013). Optimization-Based Study of Bend-Twist Coupled Rotor Blades for Passive and Integrated Passive/Active Load Alleviation. Wind. Energy.

[55] Mishnaevsky L. (2022). Root Causes and Mechanisms of Failure of Wind Turbine Blades: Overview. Materials.

[56] (2005). Standard Test Method for Mode I Interlaminar Fracture Toughness of Unidirectional Fiber-Reinforced Polymer Matrix Composite.

[57] Bhandakkar A., Kumar N., Prasad R.C., Sastry S.M.L., Bhandakkar A., Kumar N., Prasad R.C., Sastry S.M.L. (2014). Interlaminar Fracture Toughness of Epoxy Glass Fiber Fly Ash Laminate Composite. Mater. Sci. Appl..

[58] Jegley D.C., Wu K.C., Phelps J.E., Mckenney M.J., Oremont L. (2012). Structural Efficiency of Composite Struts for Aerospace Applications. J. Spacecr. Rocket..

[59] Bejan M. (2018). Strcutural Efficiency on Plastic Composites. Proc. Rom. Acad. Ser. A.

